# Characterization of the complete chloroplast genome sequence of Chinese endemic species of *Aster batangensis* (Asteraceae: Astereae) and its phylogenetic implications

**DOI:** 10.1080/23802359.2021.1990148

**Published:** 2021-10-15

**Authors:** Jiani Xie, Yuan Yong, Tian Li, Yaqi Lv, Luyao Zou, Tingyu Li, Zhuojin Li, Zhixi Fu

**Affiliations:** a School of Food and Biological Engineering, Chengdu University, Chengdu, China; b College of Chemistry and Environment Protection Engineering, Southwest Minzu University, Chengdu, China; c College of Life Sciences, Zhengzhou University, Zhengzhou, China; d College of Life Sciences, Sichuan Normal University, Chengdu, China; e Sustainable Development Research Center of Resources and Environment of Western Sichuan, Sichuan Normal University, Chengdu, China; f Institute of Application and development of plant resources, Sichuan Normal University, Chengdu, China

**Keywords:** *Aster batangensis*, complete chloroplast genome, phylogenomics analysis

## Abstract

This study was the first report complete chloroplast genome of *Aster batangensis* (Astereae: Asteraceae), the perennial herb endemic to China. The plastid genome of *Aster batangensis* include 132 unique genes, with 87 protein-coding genes, 37 tRNA genes, and 8 rRNA genes. Among these genes, 21 duplicate genes, including10 protein-coding genes, 7 tRNA genes, and 4 rRNA genes were detected. The complete genome size of *Aster batangensis* has a typical quadripartite circular structure with 152,605 bp in total length, consisting a large single copy (LSC) of 84,351 bp and a small single copy (SSC) of 18,212 bp, separated by a pair of invested repeats (IR) of 25,021 bp. The average GC content of whole plastome sequence is 37.3%, and the LSC, SSC and IR regions is 35.3%, 31.3%, and 43.0%, respectively. The phylogenetic analysis by the maximum likelihood method showed that *A. batangensis* was closely related to the other members of Astereae (e.g. *Aztecaster matudae*, *Conyza bonariensis*, *Lagenophora cuchumatanica*, *Baccharis tricuneata*, *Baccharis genistelloides*)

The genus *Aster* is one of the most diverse genera in the tribe Astereae, family Asteraceae, including about 152 species (Nesom 1994; Chen et al. 2011). The species of *Aster batangensis* Bureau and Franchet 1891 (Asteraceae, Astereae) is a perennial herb and it is endemic to southwestern China (Sichuan, Xizang, Yunnan) (Chen et al. 2011). It has ecological and medicinal value in western China. So far, the complete chloroplast genome of *A. batangensis* has not yet been published. Genetic knowledge of *A. batangensis* would provide information for protection of this wild germplasm resource. Here, we obtained the complete plastome of *A. batangensis* by Illumina sequencing technology. The complete plastome reported here will contribute to the further studies on the phylogenetic analysis of genus *Aster* and its related genera.

Fresh leaves of *A. batangensis* were collected from Bowo village (101°07′11ʺE, 28°45′′30ʺN), Muli county, Sichuan Province, China. A specimen was deposited at the botany herbarium of Sichuan Normal University, SCNU (contact person: Associate Professor, Dr. Zhixi Fu and Email: fuzx2017@sicnu.edu.cn) under the voucher number Z.X. Fu 4061. High-quality total genomic DNA was extracted from *ca*. 6 cm^2^ sections of the silica-dried leaf using improved Tiangen Plant Genomic DNA Kits, add the 4 μl RNAseA and 20 μl Proteinase K after incubated (65 °C). Total DNA was directly constructed short-insert of 150 bp in length libraries and sequenced on the Illumina Genome Analyzer (Hiseq 2000) based the manufacturer’s protocol (Illumina, San Diego, CA, USA) by ORI-GENE, Beijing. Generally, more than 3.8 Gb of data was obtained for complete chloroplast genome of *A. batangensis*. *De novo* assembled in CLC Genomic Workbench v11 (CLC Bio, Aarhus, Denmark) and consensus sequence in Geneious R11.1.5 (Biomatters Ltd., Auckland, New Zealand) with referenced chloroplast genome sequence of *Conyza bonariensis* (Accession: KX792499). The chloroplast genome were annotated using a web-based annotation program GeSeq (https://chlorobox.mpimp-golm.mpg.de/geseq.html) and editing by manual and imagining with OGDraw v1.2 (Lohse et al. 2013). We also developed the HMMER (Wheeler and Eddy 2013), tRNAscan-SE version 2.0.6 (Lowe and Eddy 1997) program which as the part of CHLOROBOX web toolbox (https://chlorobox.mpimp-golm.mpg.de/geseq.html) for checking the annotation via same reference genome. Final, the raw sequence data (SRA) and complete chloroplast genome (GenBank) of *A. batangensis* was submitted to NCBI.

The genome sequence data that support the findings of this study are openly available in GenBank of NCBI at [https://www.ncbi.nlm.nih.gov] under the accession no. MZ292735. The associated BioProject, SRA, and Bio-Sample numbers are PRJNA729213, SRP319392 and SAMN19114282 (SRS8948579) respectively. The complete genome size of *Aster batangensis* has a typical quadripartite circular structure with 152,605 bp in total length, consisting a large single-copy (LSC) of 84,351 bp and a small single-copy (SSC) of 18,212 bp, separated by a pair of inverted repeats (IR) of 25,021 bp. The average GC content of whole plastome sequence is 37.3%, and the LSC, SSC and IR regions is 35.3%, 31.3%, and 43.0%, respectively. The plastid genome of *Aster batangensis* include 132 unique genes, with 87 protein-coding genes, 37 tRNA genes, and 8 rRNA genes. Among these genes, 21 duplicate genes, including10 protein-coding genes, 7 tRNA genes, and 4 rRNA genes were detected.

To identify the phylogenetic position of *A. batangensis*, we used a total of 20 additional complete cp genomes of the family Asteraceae and two outgroup taxa to clarify the phylogenetic position of *A. batangensis* (Figure 1). All of the cp genome sequences were aligned in MAFFT (Katoh and Standley 2013). A maximum likelihood analysis based on the GTRGAMMA model was performed with RAxML method on the CIPRES (Stamatakis et al. 2008; Miller et al. 2010) using 1000 bootstrap replicates. The maximum likelihood method (ML) result showed that *A. batangensis* was closely related to the other members of Astereae (e.g. *Aztecaster matudae*, *Conyza bonariensis*, *Lagenophora cuchumatanica*, *Baccharis tricuneata*, *Baccharis genistelloides*) (Bootstrap support = 100, Figure 1). The complete cp genome sequence of *A. batangensis* will be the valuable resource for future studies on taxonomy and phylogeny of family Asteraceae and provides useful molecular data for further phylogenetic and evolutionary analysis.

**Figure 1.**
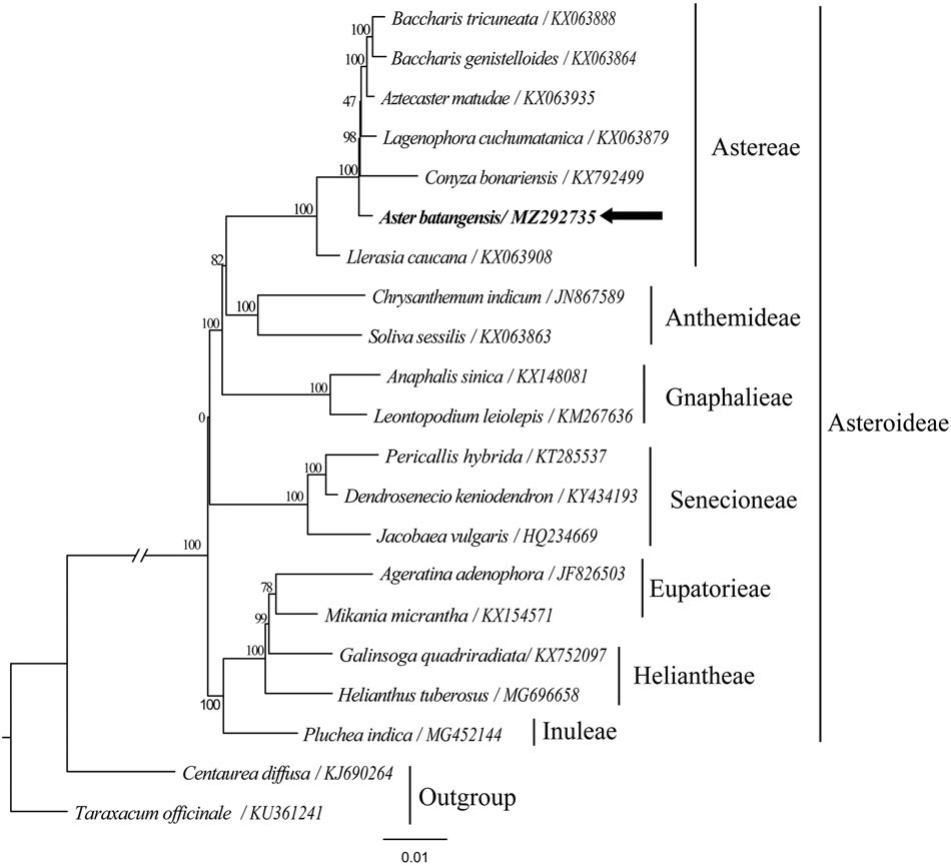
A maximum-likelihood (ML) tree inferred from 31 chloroplast genomes in Asteraceae (the support value are indicated on the branches). The position of *Aster batangensis* is in bold.

## Data Availability

The genome sequence data that support the findings of this study are openly available in GenBank of NCBI at (https://www.ncbi.nlm.nih.gov/) under the accession no. MZ292735. The associated BioProject, SRA, and Bio-Sample numbers are RJNA729213, SRP319392 and SAMN19114282 (SRS8948579) respectively.
